# Supplementary material for: A structure of substrate-bound Synaptojanin1 provides new insights in its mechanism and the effect of disease mutations
Source: eLife. 2020 Dec 22;9:e64922. doi: 10.7554/eLife.64922 (PMC7781601; doi:10.7554/eLife.64922)
Supplement: Supplementary file 1. [file elife-64922-supp1.docx]

**Supplementary file 1A: Comparison of the 5PPase domain of human Synj1 (Synj1_528-873_) with the corresponding 5PPase domain of the other human inositol polyphosphate 5-phosphatases and SPSynj.** The root-mean-square deviation (rmsd) after superposition of the structures was determined using CCP4 SUPERPOSE. Furthermore, the sequence identity was determined using Clustal Omega.

| **5PPase domain of** | **PDB code** | **Rmsd** | **Sequence identity** |
| --- | --- | --- | --- |
| **INPP5B** | 3N9V | 1.15 Å  (for 273 superposed residues) | 39.60% |
| **SHIP2** | 3NR8 | 1.24 Å  (for 269 superposed residues) | 29.24% |
| **OCRL** | 4CMN | 1.22 Å  (for 283 superposed residues) | 38.26% |
| **INPP5E or Pharbin** | 2XSW | 1.35 Å  (for 276 superposed residues) | 34.36% |
| **SPSynj** | 1I9Y | 1.20 Å  (for 281 superposed residues) | 41.95% |
| **SHIP1** | / | / | 31.56 |
| **Synj2** | / | / | 63.01% |
| **INPP5J or PIPP** | / | / | 23.15% |
| **SKIP** | / | / | 29.94% |
| **INPP5A** | / | / | 21.48% |

**Supplementary file 1B: Residues interacting with the diC8-PI(3,4,5)P_3_ substrate in Synj1_528-873_ and the corresponding residues in the other nine human 5PPases and SPSynj.** Completely conserved residues are written in white with a red background and similar residues are written in red.

|  | **Synj1** | **Synj2** | **SPSynj** | **INPP5B** | **OCRL** | **SHIP1** | **SHIP2** | **INPP5A** | **INPP5E** | **INPP5J** | **SKIP** |
| --- | --- | --- | --- | --- | --- | --- | --- | --- | --- | --- | --- |
| **1P interacting residues** | N668  **K669** | N664  **K665** | N678  **K679** | N379  **K380** | N354  **K355** | N519  **K520** | N540  **K541** | R162  **K163** | T403  **K404** | N540  **K541** | N128  **K129** |
| **4P interacting residues** | K798  R800  Y784 | K794  R796  Y780 | K808  R810  Y794 | K516  R518  Y502 | K491  R493  Y477 | K665  N667  Y644 | R682  N684  Y661 | N340  R342  Y326 | K548  R550  Y534 | K673  R675  F659 | K261  R263  Y247 |
| **5P interacting residues** | **H689**  **R734**  **H859** | **H685**  **R730**  **H855** | **H699**  **R744**  **H839** | **H400**  **R451**  **H549** | **H375**  **R426**  **H524** | **H540**  **R591**  **H701** | **H561**  **R611**  **H718** | **H183**  **R235**  **H384** | **H424**  **R481**  **H584** | **H561**  **R608**  **H722** | **H149**  **R196**  **H311** |
| **Residues involved in nucleophilic attack** | **D730**  **N732** | **D726**  **N728** | **D740**  **N742** | **D447**  **N449** | **D422**  **N424** | **D587**  **N589** | **D607**  **N609** | **D231**  **N233** | **D477**  **N479** | **D604**  **N606** | **D192**  **N194** |
| **Mg^2+^-coordinating**  **residues** | N543  **E591** | N540  **E587** | N568  **E597** | N275  **E303** | N250  **E278** | G414  **E453** | G434  **E473** | G19  **E54** | Q309  **E340** | G434  **E466** | A24  **E54** |
